# Comprehensive transthoracic echocardiographic evaluation of doxorubicin-induced cardiotoxicity: a multimodal imaging approach in an animal model

**DOI:** 10.1093/ehjimp/qyaf006

**Published:** 2025-01-10

**Authors:** Sibren Haesen, Lisa Steegen, Dorien Deluyker, Virginie Bito

**Affiliations:** UHasselt, Cardio & Organ Systems (COST), BIOMED, Agoralaan, Diepenbeek 3590, Belgium; UHasselt, Cardio & Organ Systems (COST), BIOMED, Agoralaan, Diepenbeek 3590, Belgium; UHasselt, Cardio & Organ Systems (COST), BIOMED, Agoralaan, Diepenbeek 3590, Belgium; UHasselt, Cardio & Organ Systems (COST), BIOMED, Agoralaan, Diepenbeek 3590, Belgium

**Keywords:** doxorubicin, cardiotoxicity, animal model, echocardiography

## Abstract

**Aims:**

Anthracycline-induced cardiotoxicity has high incidence rates and causes significant mortality among cancer survivors. Damage to myocardial tissue leads to left ventricular (LV) dilation with systolic dysfunction, typically assessed through echocardiographic measurement of LV ejection fraction (LVEF) and volumes. Early detection is crucial for improving patient outcomes. We aimed to evaluate cardiotoxicity progression and diagnostic performance of different echocardiographic modalities in an animal model.

**Methods and results:**

Female Sprague Dawley rats received either intravenous doxorubicin (DOX) injections weekly for 8 weeks (2 mg/kg/week) or saline (control). Transthoracic LV echocardiography was performed before treatment and at 4, 6, and 8 weeks in the treatment course. Two researchers performed and evaluated M-mode, B-mode, and four-dimensional (4D) echocardiography. Bland–Altman plots were created to show the bias and limits of agreement when comparing echocardiographic modalities. Simple linear regression and Pearson correlation were applied to evaluate interobserver variability. Six weeks after the first DOX injection, LVEF, radial LV fractional shortening, LV end-systolic volume, and LV end-diastolic volume were significantly reduced compared with baseline. LV dysfunction and dilation became more pronounced after 8 weeks of DOX treatment. For all parameters, 4D- and M-mode showed the lowest bias and narrowest limits of agreement. The correlation between the researchers’ measurements was strong for most parameters.

**Conclusion:**

Our rat model of DOX-induced cardiotoxicity demonstrates that volumetric changes are more pronounced. Both 4D- and M-mode imaging techniques proved effective and reliable compared with the standard B-mode approach, with minimal interobserver variability, indicating strong reproducibility.

## Graphical Abstract

**Graphical Abstract:**
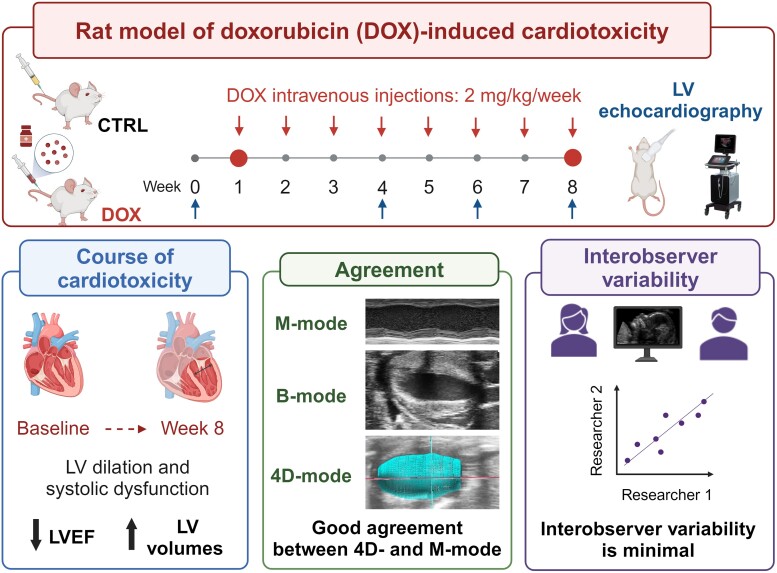
4D, four-dimensional; CTRL, control; DOX, doxorubicin; LV, left ventricle; LVEF, left ventricular ejection fraction. Created with BioRender.com.

## Introduction

Anthracyclines like doxorubicin (DOX) rank among the most potent chemotherapeutic agents extensively used to treat various cancers, including breast cancer, lymphomas, sarcomas, and leukaemias. Despite its efficacy, DOX is known for its cardiotoxicity. The pathogenesis of DOX-induced cardiotoxicity involves several mechanisms, with mitochondria identified as the primary subcellar targets.^1^ Cardiac damage starts from the first exposure, but the extent is predominantly determined by the cumulative dose of DOX administered.^2^ Upon DOX exposure, the heart undergoes structural changes characterized by left ventricular (LV) dilation and a weakened heart muscle with decreased systolic function. This adverse phenotype potentially progresses to heart failure and poses a significant clinical challenge, contributing to high morbidity and mortality among DOX-treated cancer patients and survivors.^3^ Given the importance of preserving cardiac function during cancer treatment, various strategies to mitigate anthracycline-induced cardiotoxicity have been explored.^4^ However, these efforts have not yet resulted in a universally helpful strategy. The efficacy of cardioprotective strategies decreases with increasing time between damage onset and the initiation of therapy. Early detection of cardiotoxicity in a subclinical stage could prevent cardiomyocyte loss and limit the development of irreversible cardiac injury.^5^ Therefore, early detection and accurate monitoring of DOX-induced cardiotoxicity are crucial to improving patient prognosis. Two-dimensional echocardiography (2DE) allows the detection of cardiotoxicity by measuring LV ejection fraction (LVEF). DOX-induced cardiotoxicity has long been defined as a decrease of LVEF by >10% points from baseline to an LVEF < 55%.^6^ Although conventional 2DE is the most applied tool, preclinical studies indicate that more innovative imaging methods are needed for cardiac assessment. Three- (3DE) or four-dimensional echocardiography (4DE) is more accurate, demonstrates less variation, and correlates better with magnetic resonance imaging than traditional 2DE.^7^ In addition, differences in the accuracy of various echocardiographic modalities (e.g. M-mode and B-mode) and interobserver consistency can lead to misinterpreted data. Preclinical studies show conflicting results regarding which parameters change first after DOX treatment. In addition, there is a lack of studies comparing the effectiveness of different echocardiographic modalities in detecting DOX-induced cardiotoxicity in animal models. Our study aimed to evaluate the onset and progression of cardiac injury and changes in echocardiography parameters over time in a rat model of DOX-induced cardiotoxicity. Moreover, we assessed the diagnostic performance of different echocardiographic modalities and assessed interobserver consistency.

## Methods

### Animal model and study design

All animal experiments follow the EU Directive 2010/63/EU for animal testing and are approved by the local ethical committee (Ethical Commission for Animal Experimentation, UHasselt, Diepenbeek, Belgium, ID 201942 and ID 202154). Animals were group-housed in a standard cage with cage enrichment at the conventional animal facility of UHasselt and fed a standard pellet diet with water available *ad libitum*. The environmental conditions were rigorously controlled (i.e. 22°C temperature and 22–24% humidity). Six-week-old female Sprague Dawley rats (Janvier Labs, Le Genest-Saint-Isle, France) were randomly allocated into two groups. The first group received DOX (2 mg/kg, *n* = 14, Accord Healthcare B.V., Utrecht, The Netherlands), intravenously injected weekly for 8 weeks (16 mg/kg cumulative dose). The model adheres to the current guidelines.^8^ The second group received an equal volume of 0.9% saline as a control group (CTRL, *n* = 14). Echocardiography was performed at baseline and 4, 6, and 8 weeks after the first injection. The study was conducted in two cohorts, with echocardiography performed in all animals at baseline and Week 8. Echocardiography at Week 4 and Week 6 was conducted in part of the animals (*n* = 7 at both time points). Rats were euthanized with an overdose of sodium pentobarbital (150 mg/kg intraperitoneal, Dolethal, Vetoquinol, Aartselaar, Belgium) in Week 9 of the study.

### Echocardiography

LV transthoracic echocardiography was performed using a Vevo® 3100 high-resolution imaging system with a 21 MHz MX250 transducer (FUJIFILM VisualSonics, Inc., Amsterdam, The Netherlands) as described before.^9^ Parasternal long-axis images were acquired in single-plane B-mode with ECG (electrocardiogram)-gated kilohertz visualization to measure longitudinal LV fractional shortening (LVFS), LV end-systolic volume (LVESV), and LV end-diastolic volume (LVEDV). Pulsed-wave (PW) Doppler and tissue Doppler modes were used to measure the ratio of peak flow velocity in early vs. late filling (E/A) and of peak mitral flow vs. annular velocity (E/E′). Parasternal short-axis images were acquired in M-mode to measure radial LVFS. 4DE was performed to measure LVESV, LVEDV, LVEF, and LV cardiac output (LVCO). All analyses were performed using Vevo® LAB (Vevo® LAB software, version 5.6.1, FUJIFILM VisualSonics, Inc.). Two independent and blinded researchers analysed the echocardiographic images.

### Strain measurement

To perform strain analysis of the LV, mid-ventricular parasternal short- and long-axis B-mode cine loops were imported into the Vevo® Strain software (version 5.6.1, FUJIFILM VisualSonics, Inc.). The most optimal cardiac cycles were selected to assess LV global circumferential strain (LVGCS, short-axis), LV peak radial strain (long-axis), and LV global longitudinal strain (LVGLS, long-axis). The endocardial border was manually traced, and simultaneous epicardial tracing was performed automatically to obtain 48 sampling points, dividing the LV into six myocardial segments. In each segment, peak strain was analysed.

Random missing values of echocardiographic parameters are due to poor image quality or deceased animals.

### Statistical analysis

Statistical analysis was performed using GraphPad Prism (GraphPad software, version 10.2.1). The normal data distribution was tested with the D’Agostino and Pearson normality test. Repeated measures mixed-effects analysis was performed for parameters assessed at multiple time points. When data were not normally distributed, a Mann–Whitney test was used to compare values between two time points. The parametric one-way ANOVA test was performed to compare parameters measured with different echocardiographic modalities at Week 8. Bonferroni *post hoc* tests were performed for multiple comparisons. The Bland–Altman comparison method evaluated the agreement between the echocardiographic modalities by plotting the difference in the measurements of the two modalities against their average. The mean of the differences (bias) and 95% limits of agreement were calculated. Measurements of two researchers were compared with parametric unpaired *t*-tests to evaluate interobserver variability. Simple linear regression models were applied to correlate the measurements. Pearson correlation coefficients (*r*) were determined. Outliers were identified using the ROUT method with a maximum desired false discovery rate of 1%, with this criterion established *a priori*. Data are expressed as the mean ± SEM. *P* < 0.05 was considered statistically significant.

## Results

### After 6 weeks, DOX affects LV systolic function, strain, and volumes

**Figure 1:**
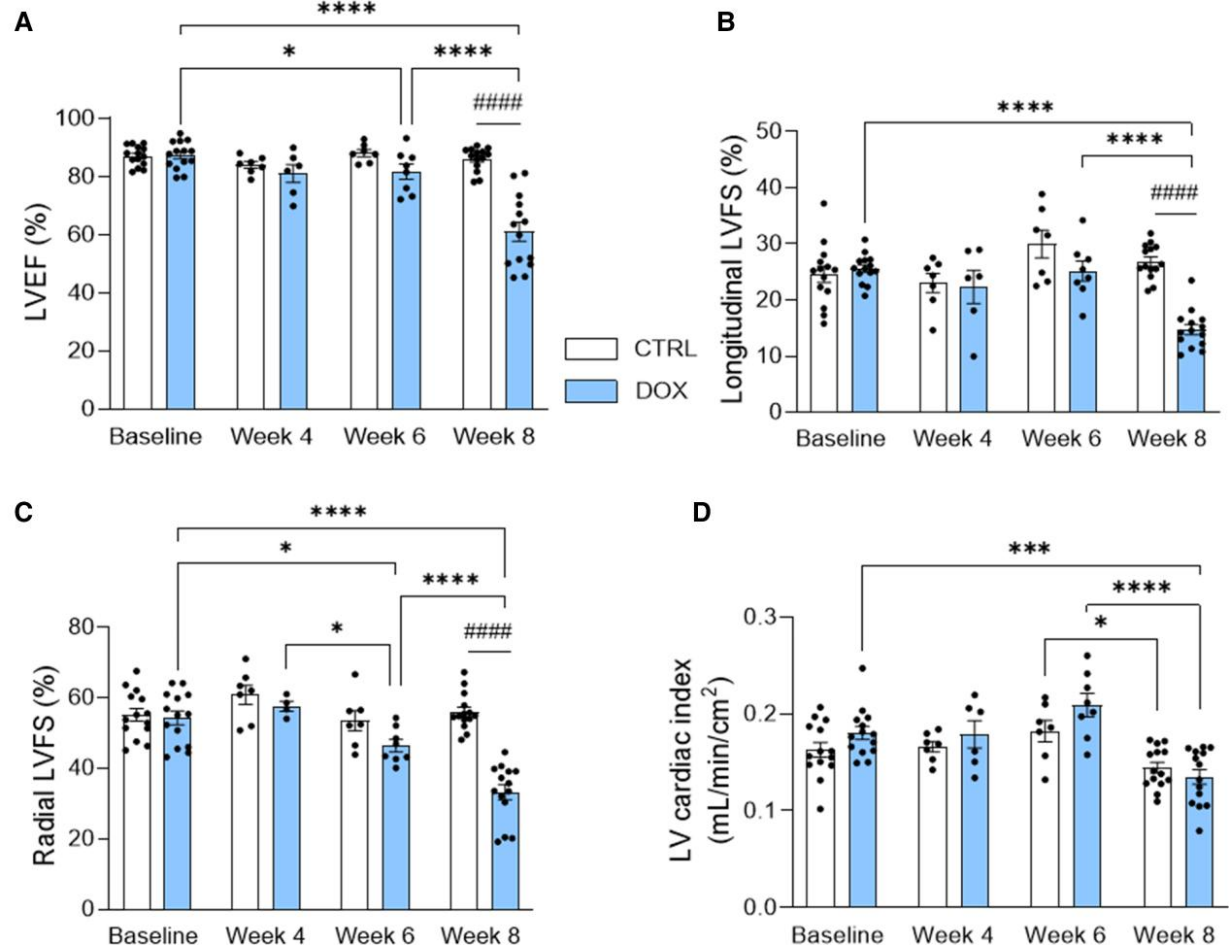
LV systolic function over time. LVEF (*A*), longitudinal LVFS (*B*), radial LVFS (*C*), and LV cardiac index (*D*) measured in CTRL and DOX animals (both *n* = 14). The LV cardiac index is calculated by normalizing LVCO to body surface area. Data are shown as mean ± SEM. For CTRL: *n* = 14 (baseline, Week 8) and *n* = 7 (Week 4, Week 6). For DOX: *n* = 14 (baseline, Week 8), *n* = 4/6 (Week 4), and *n* = 8 (Week 6). *P < 0.05, ***P < 0.001, and ****P < 0.0001. ^####^*P* < 0.0001. CTRL, control; DOX, doxorubicin; LV, left ventricular; LVEF, left ventricular ejection fraction; LVFS, left ventricular fractional shortening.

Echocardiography of the LV was performed at baseline and after 4, 6, and 8 weeks of DOX treatment to measure parameters of LV systolic function, diastolic function, strain, and volumes. 4D-, B-, and M-modes were used to measure LVEF and LV cardiac index, longitudinal LVFS, and radial LVFS, respectively (Supplementary data online, *Tables S1* and *S2*). Four weeks of DOX treatment did not affect systolic function compared with baseline and CTRL (*Figure 1*). At Week 6, DOX-treated rats showed significantly reduced LVEF and radial LVFS compared with baseline (LVEF: −7% and radial LVFS: −14%; both *P* < 0.05) (*Figure 1A* and *C*). Longitudinal LVFS and LV cardiac index were not significantly affected at this time point (*Figure 1B* and *D*). From Week 6 onward and after 8 weeks of DOX treatment, all parameters of systolic function were significantly reduced compared with baseline (LVEF: −30%; *P* < 0.0001, longitudinal LVFS: −43%; *P* < 0.0001, radial LVFS: −39%; *P* < 0.0001, LV cardiac index: −25%; *P* < 0.001). LV cardiac index significantly differed for CTRL rats between 6 and 8 weeks (*P* < 0.05). Significant differences between the CTRL and DOX groups were found only for Week 8, with LVEF, longitudinal LVFS, and radial LVFS significantly decreased in DOX rats (all *P* < 0.0001). Representative echocardiographic images obtained at Week 8 can be found in supplement (Supplementary data online, *Figure S1*).

**Figure 2:**
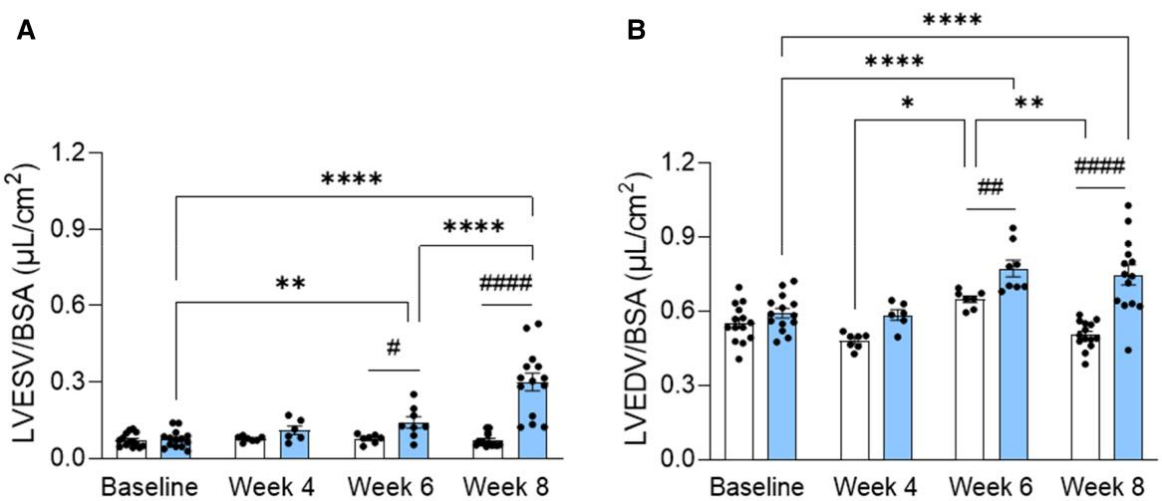
LV volumes over time. LVESV (*A*) and LVEDV (*B*) measured with 4D-mode in CTRL and DOX animals (both *n* = 14). Data are shown as mean ± SEM. For CTRL: *n* = 14 (baseline, Week 8) and *n* = 7 (Week 4, Week 6). For DOX: *n* = 14 (baseline, Week 8), *n* = 6 (Week 4), and *n* = 8 (Week 6). *P < 0.05, **P < 0.01, and ****P < 0.0001. ^##^*P* < 0.01 and ^####^*P* < 0.0001. BSA, body surface area; CTRL, control; DOX, doxorubicin; LVESV, left ventricular end-systolic volume; LVEDV, left ventricular end-diastolic volume.

Volumetric measurements were performed with 4DE and are shown in *Figure 2*. Compared with baseline, DOX-treated rats showed no change in LV volumes at Week 4, while a significant increase in LVESV (+87%; *P* < 0.01) and LVEDV (+30%; *P* < 0.0001) was observed at Week 6, which was further increased at Week 8 for LVESV (+296%; *P* < 0.0001 vs. baseline) (*Figure 2A* and *B*). Also, in CTRL rats, LVEDV significantly differed between time points. LVESV and LVEDV were significantly different between CTRL and DOX groups at Week 6 (*P* < 0.05 and *P* < 0.01, respectively) and were more pronounced at Week 8 (*P* < 0.0001).

Diastolic function parameters remained unchanged in the DOX group at all time points and were similar to the CTRL group (see Supplementary data online, *Figure S2A* and *B*). CTRL rats showed a significant −E/E′ reduction at Week 4 and Week 8 compared with baseline (*P* < 0.05 and *P* < 0.01; Supplementary data online, *Figure S2B*). Furthermore, survival for CTRL and DOX groups represented as a Kaplan-Meier plot in supplement (Supplementary data online, *Figure S3*).

Strain measurements were performed with B-mode and are shown in Supplementary data online, *Figure S4*. GCS shows a significant decrease in DOX animals between baseline and Weeks 4 or 6 (*P* < 0.001 and *P* < 0.0001) (see Supplementary data online, *Figure S4B*). Four and 6 weeks of DOX treatment did not affect GLS and LV peak radial strain compared with baseline and CTRL (see Supplementary data online, *Figure S4A* and *C*). At Week 8, DOX-treated rats showed significantly reduced GLS, GCS, and LV peak radial strain compared with baseline (GLS: −36%; *P* < 0.0001, GCS: −36%; *P* < 0.0001, peak radial strain: −48%; *P* < 0.0001) (see Supplementary data online, *Figure S4*). GLS and GCS significantly differed for DOX-treated rats between 4 and 8 weeks (both *P* < 0.05), and GLS was more pronounced between 6 and 8 weeks (*P* < 0.0001) (see Supplementary data online, *Figure S4A* and *B*). DOX-treated rats significantly differed for GCS and LV peak radial strain between Weeks 6 and 8 (GCS *P* < 0.05 and LV peak radial strain *P* < 0.01) (see Supplementary data online, *Figure S4B* and *C*). Significant differences between the CTRL and DOX groups were found for Weeks 6 and 8 with GCS (*P* < 0.01 and *P* < 0.0001) and for Week 8 with GLS and LV peak radial strain decreased in DOX rats (both *P* < 0.0001) (see Supplementary data online, *Figure S4*).

### 4D- and M-modes show the best agreement to evaluate LV systolic function and volumes

**Figure 3:**
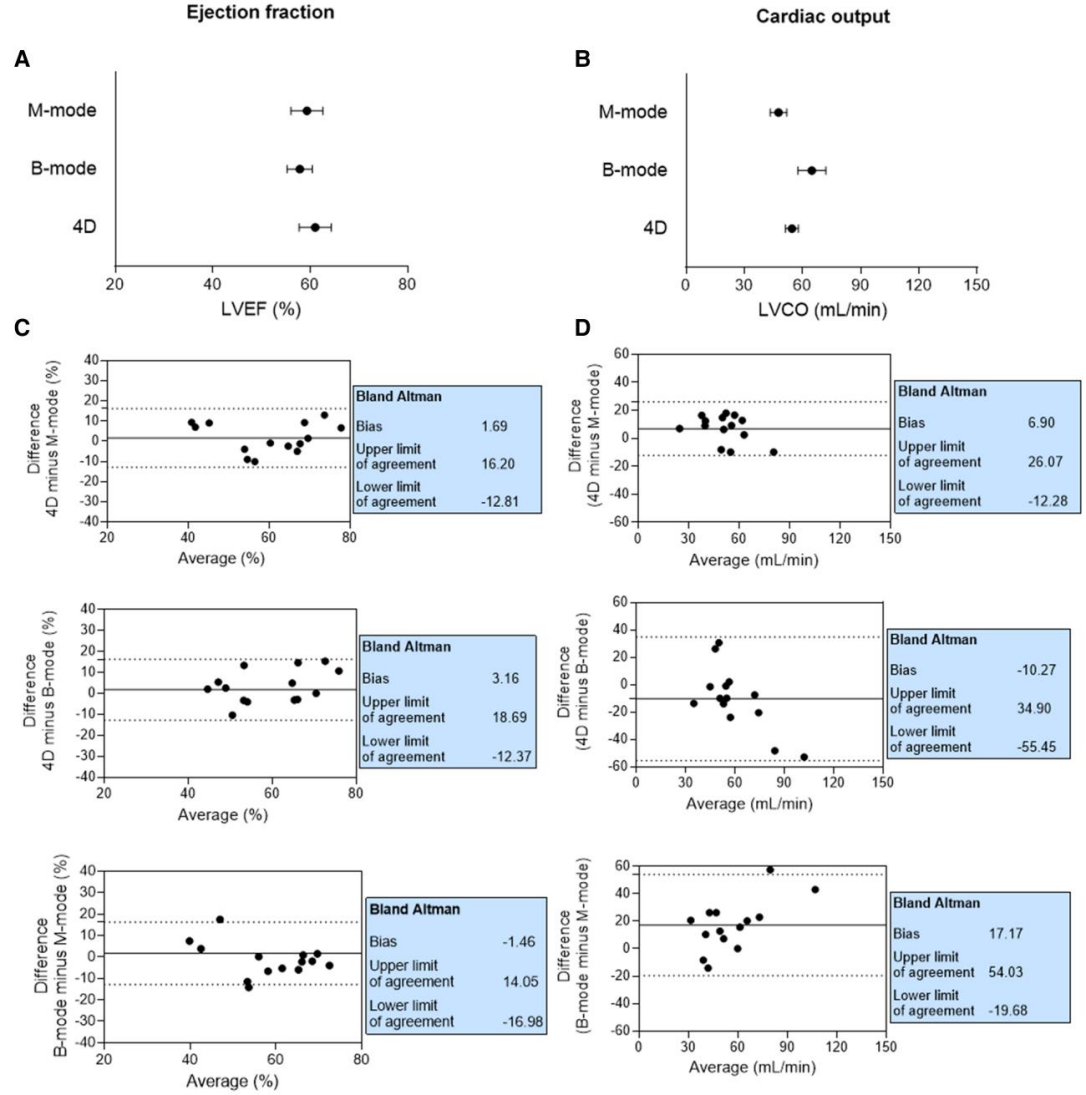
Agreement between echocardiographic modalities for assessing LV systolic function. Comparison of LVEF (*A*) and LVCO (*B*) measured after 8 weeks of treatment with different echocardiographic modalities in DOX animals (*n* = 14). Bland–Altman comparisons of different modes for LVEF (*C*) and LVCO (*D*). For (*A*) and (*B*), data are shown as mean ± SEM. LVEF, left ventricular ejection fraction; 4D, four-dimensional; LVCO, left ventricular cardiac output.

Multimodal echocardiography assessed LV systolic function and volumes in DOX-treated rats at Week 8. LVEF and LVCO were not significantly different between modalities (*Figure 3A* and *B*). The variance of LVEF was similar with each modality, while for LVCO, it was markedly greater with B-mode compared with other modalities. For LVEF, the bias was low and the limits of agreement were narrow for all comparisons, with 4D- and M-modes showing the best agreement [bias 1.69 (−12.81; 16.20)] (*Figure 3C*). Furthermore, the agreement for LVCO was best between 4D- and M-modes [bias 6.90 (−12.28; 66.07)], with a notable difference from the other comparisons (*Figure 3D*). When including B-mode in the comparison, the bias increased and the agreement limits broadened.

**Figure 4:**
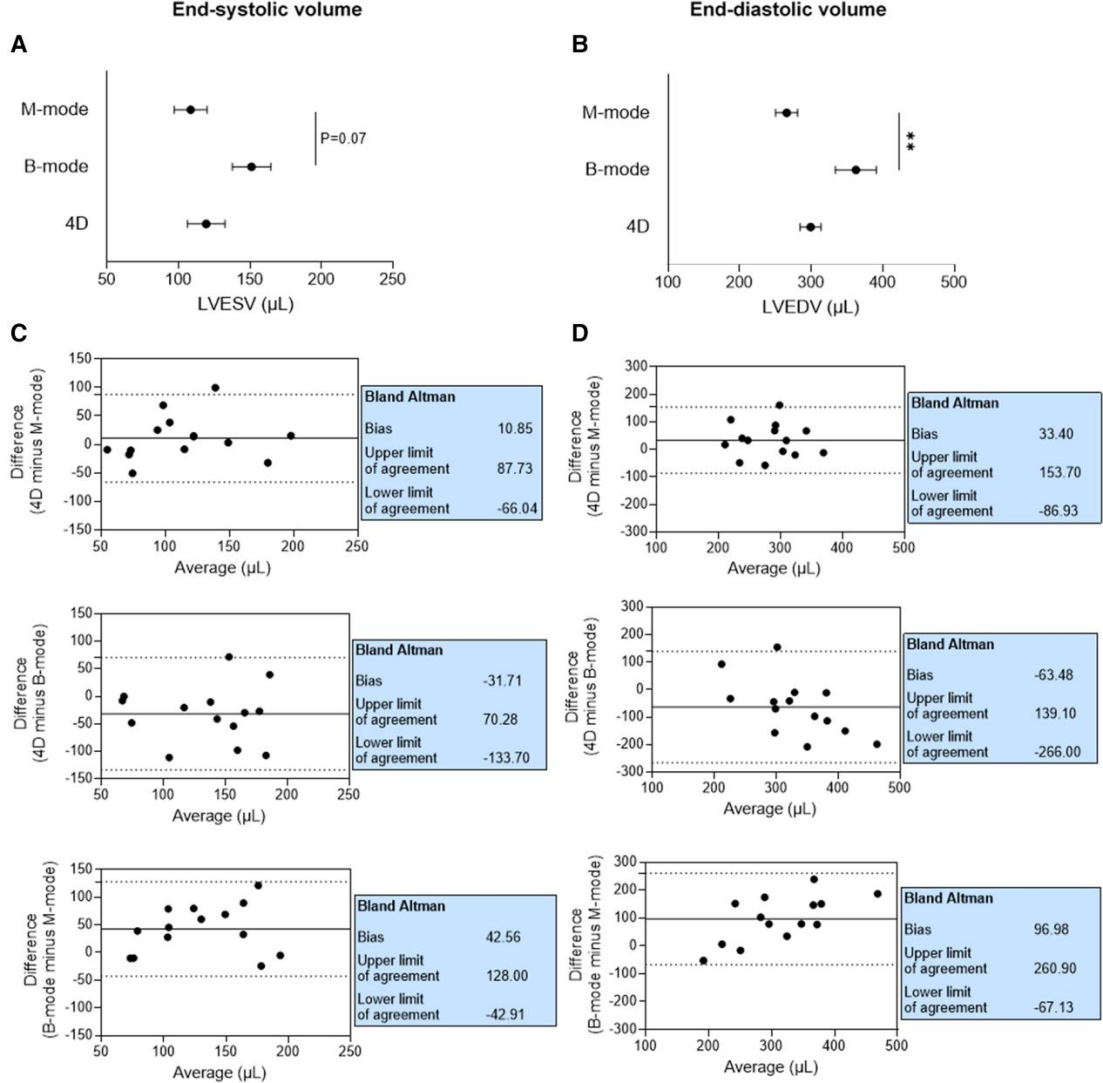
Agreement between echocardiographic modalities for assessing LV systolic volumes. Comparison of LVESV (*A*) and LVEDV (*B*) measured after 8 weeks of treatment with different echocardiographic modalities in DOX animals (*n* = 14). Bland–Altman comparisons of different modes for LVEF (*C*) and LVCO (*D*). For (*A*) and (*B*), data are shown as mean ± SEM. **P < 0.01. 4D, four-dimensional; LVESV, left ventricular end-systolic volume; LVEDV, left ventricular end-diastolic volume.

Regarding volumes, LVEDV was significantly higher when measured with B-mode compared with M-mode (*P* < 0.01), and a similar trend was observed for LVESV (*P* = 0.07) (*Figure 4A* and *B*). Again, Bland–Altman plots indicated the lowest bias and narrowest limits of agreement for comparing 4D- and M-mode (*Figure 4C* and *D*).

### The interobserver variability of echocardiographic measurements is minimal

**Figure 5:**
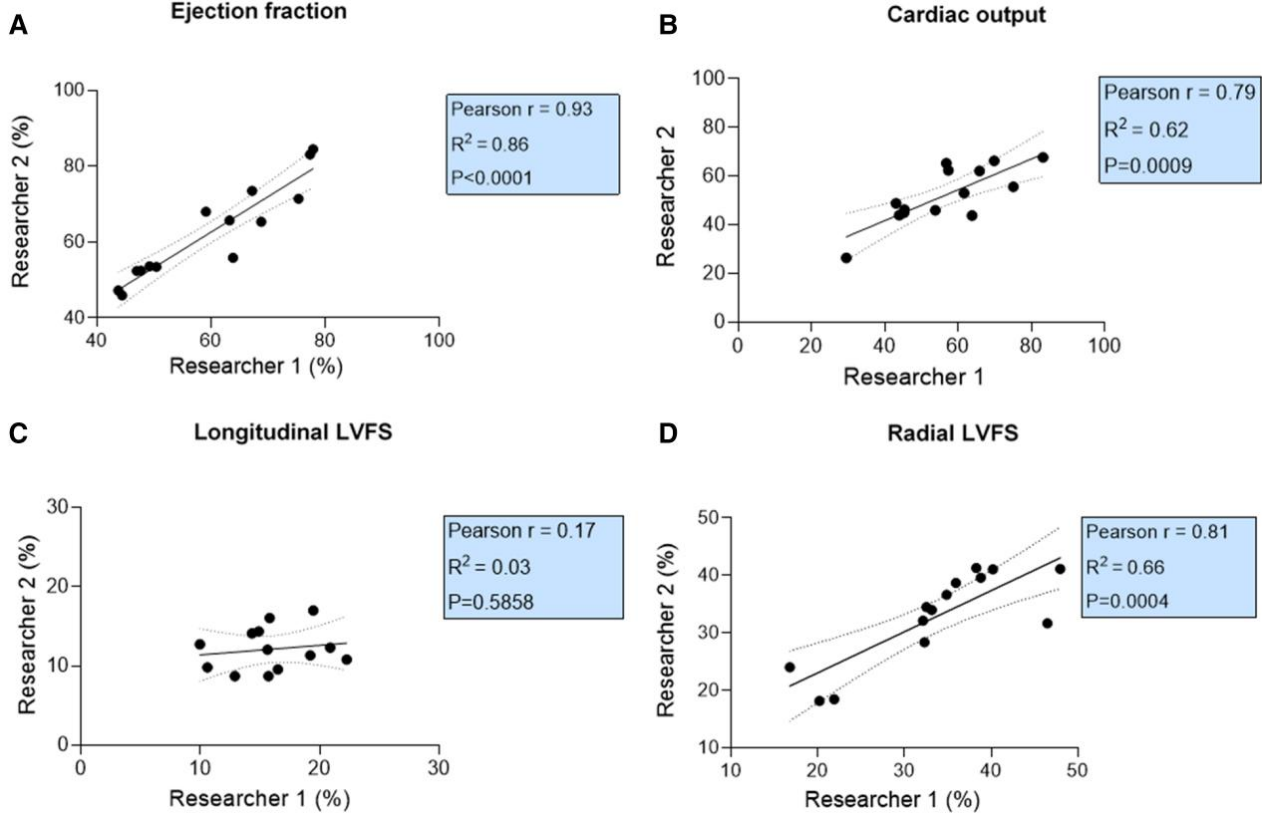
Interobserver variability for LV systolic function. Correlation between the measurements of both researchers for LVEF (*A*), LVCO (*B*), longitudinal LVFS (*C*), and radial LVFS (*D*) in DOX animals after 8 weeks of treatment (*n* = 14). LVEF, left ventricular ejection fraction; LVCO, left ventricular cardiac output; LVFS, left ventricular fractional shortening.

As shown in *Figure 5*, both researchers showed a strong correlation for LVEF (Pearson *r* = 0.93; *P* < 0.0001) and LVCO (Pearson *r* = 0.79; *P* = 0.0009) (*Figure 5A* and *B*). In contrast, longitudinal LVFS was weakly correlated between both researchers (Pearson *r* = 0.17), suggesting substantial interobserver variability (*Figure 5C*). For radial LVFS, a strong correlation was observed (Pearson *r* = 0.81; *P* = 0.0004) (*Figure 5D*).

**Figure 6:**
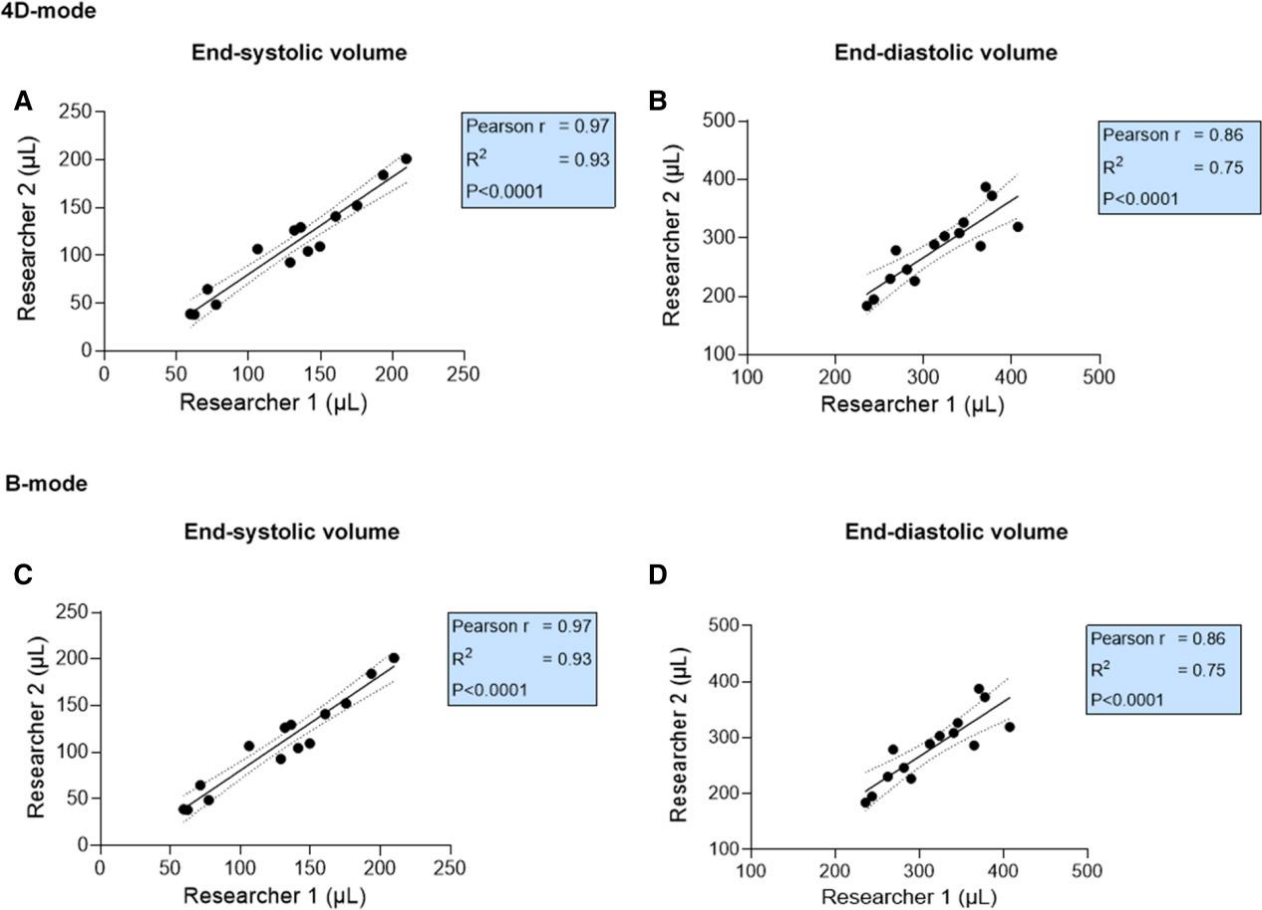
Interobserver variability for LV systolic volumes. Correlation between both researchers for LVESV and LVEDV measured in 4D-mode (*A* and *B*) and B-mode (*C* and *D*) in DOX animals after 8 weeks of treatment (*n* = 14). 4D, four-dimensional; LVESV, left ventricular end-systolic volume; LVEDV, left ventricular end-diastolic volume.

Regarding volumes measured with 4DE, both researchers showed a strong correlation for LVESV (Pearson *r* = 0.97; *P* < 0.0001) (*Figure 6A*) and LVEDV (Pearson *r* = 0.86; *P* < 0.0001) (*Figure 6B*). In contrast, the correlation was weak for LVESV measured with B-mode (Pearson *r* = 0.43) (*Figure 6C*). For LVEDV, the correlation remains consistent with that observed for 4DE (*Figure 6D*).

## Discussion

In this study, we conducted a comprehensive echocardiographic evaluation of a rat model of DOX-induced cardiotoxicity, demonstrating the superior reproducibility of 4D- and M-mode over B-mode for assessing disease progression.

### Volume changes are more pronounced following DOX treatment

The prognosis of a cancer patient suffering from or at high risk of developing anthracycline-induced cardiac injury is highly dependent on early discovery.^10^ While clinical symptoms may not appear until cardiac reserves are depleted, mild dysfunction may exist. Transthoracic echocardiography is a non-invasive, safe, and reliable tool for monitoring cardiac function in rodent cardiotoxicity models.^11^ In our study, we confirmed the onset of cardiotoxicity starting at 6 weeks post-injection (i.e. cumulative dose of 12 mg/kg), characterized by elevated LV volumes, impaired LV systolic function parameters, and strain parameters, resembling the clinical phenotype seen in DOX-treated patients. While volumetric measurements uniformly changed at Week 6, not all systolic function parameters showed early alterations as LVEF often fails to demonstrate early myocardial damage.^7^ Growing evidence suggests that strain may be more sensitive to detect early myocardial changes.^12^ Systematic reviews indicate that a decline in GLS precedes LVEF decline, is associated with higher cardiotoxicity risk, and is more accurate in detecting early LV dysfunction.^13,14^ In contrast, discrepancies exist in preclinical rodent studies due to variations in measurement techniques and animal models.^15,16^ Notably, evidence suggests that GCS may precede GLS in detecting early chemotherapy-induced cardiotoxicity, particularly under conditions of spherical remodelling.^17^ In our study, GCS demonstrated superior sensitivity, detecting cardiotoxicity after 4 weeks of the first DOX injection compared with 8 weeks for GLS. This highlights the complementary role of GCS, particularly in capturing circumferential shortening and providing a more sensitive and comprehensive assessment of subclinical cardiac dysfunction. In addition, given the increased severity of systolic dysfunction and LV dilation at Week 8 in this study, follow-up studies are warranted to assess further progression of cardiotoxicity in our model. Multiple studies have reported similar systolic and volumetric impairment in rodents treated with DOX. While Chan *et al*.^18^ showed a significant decrease in LVFS and LVEF and an increase in LVESV in mice treated with a cumulative dose of 24 mg/kg DOX relative to CTRL, others reported similar decreases in systolic function parameters at lower cumulative DOX doses.^19–21^ However, in some studies, systolic function was unaltered at these doses. While Ohlig *et al.*^22^ showed no significant changes in LVEF and LVFS after 18 mg/kg DOX, Chakouri *et al.*^23^ only showed a decrease in LVEF and LVFS 1 month after the last injection in rats treated with a cumulative dose of 12.5 mg/kg DOX. Although DOX-induced cardiotoxicity is primarily associated with the deterioration of systolic function, it can also affect diastolic function,^24^ which was not observed in our study. Variations in cardiac load, cardiac rhythm, age, and differences in measurement techniques may account for the conflicting results in studies.^6^ Discrepancies across studies may also be explained by variations in the treatment regimen (i.e. cumulative dose and duration). Noteworthy, we performed 4DE to evaluate the progressive deterioration of cardiac function over time whereas other studies mainly relied on M- or B-mode echocardiography. Stegmann *et al.*^25^ demonstrated that 4DE is as precise as 4D CMR and highly reproducible, emphasizing its status as the preferred method for cardiac assessment. Finally, radial LVFS was measured using M-mode imaging, while longitudinal LVFS was assessed with B-mode imaging. In our model, B-mode has proven to be the least ideal imaging modality, while M-mode would be a better choice for our model (and 4D imaging being the optimal choice). Therefore, it is likely that the earlier detection of changes in radial LVFS may result from the higher sensitivity of M-mode imaging compared with B-mode, rather than a true mechanistic difference.

### The reproducibility of echocardiography: B-mode echocardiography is not as suitable to evaluate DOX-induced cardiotoxicity in a rat model

Echocardiography offers a reliable, cost-effective, and widely accessible technique for evaluating cardiac function in humans and small animals. Multiple echocardiographic modalities are available, including M-mode, B-mode, and 3D/4D-modes.^26^ However, a major limitation of M- and B-mode is that they depend on geometric assumptions to reconstruct the physiologically irregular shape of the heart.^27^ Moreover, small errors during the analyses have major implications for the accuracy of volumetric calculations. Therefore, 3D/4D-mode echocardiography should be the most reliable method and the prevailing choice for functional and volumetric measurements of the LV as it does not rely on geometrical assumptions. 4DE also strongly agrees with CMR, confirmed in preclinical studies with rodents^25,28^ and humans.^29,30^ However, a survey from the European Association of Cardiovascular Imaging in 2020 revealed that using two-dimensional (2D) rather than 3D/4D echocardiography remains frequently used in the clinic.^31^ Moreover, echocardiography can show variable reproducibility due to different echocardiographic modalities or operators.^32^ In addition, direct comparisons of 4D-, B-, and M-mode for systolic, diastolic, and volumetric parameters, particularly in preclinical models of DOX-induced cardiotoxicity, are limited. Our study is the first to present a head-to-head comparison of these modalities. We used the Bland–Altman comparison method to evaluate agreement and assessed interobserver variability by correlating the measurements by two researchers as recommended by Bunting *et al.*^32^ Our study shows that all echocardiographic modalities provide comparable results when assessing LV systolic function parameters, with Bland–Altman plots demonstrating low bias and narrow limits of agreement. However, B- and M-mode showed low agreement for volumetric measurements, and using B-mode generally worsened agreements. Indeed, 4D- and M-mode echocardiography showed the best agreement for all parameters of LV systolic function and volumes, with the lowest bias and narrowest limits of agreement. These findings indicate that B-mode data are less reliable for monitoring DOX-induced cardiotoxicity than 4D- and M-mode data. Previous studies have demonstrated that 4DE significantly enhances the accuracy and reproducibility of LV function quantification compared with B-mode echocardiography,^25,33^ as it minimizes motion artefacts through ECG and respiratory gating. Although guidelines indicate that B-mode is preferable over M-mode echocardiography,^15^ our results demonstrate less variability and a higher reproducibility for M-mode than B-mode. This could be explained by the fact that no modified Simpson’s rule was performed. We acquired single-plane B-mode images from a parasternal long-axis view. However, Zacchigna *et al.*^15^ also proposed that the modified Simpson’s method allows measurements of LV volumes with higher accuracy. This method combines B-mode images from a parasternal long-axis view with images from three short axes (i.e. base, mid, and apex) to assemble a 3D reconstruction of the LV. It is more accurate in quantifying volumes in pathological conditions. Therefore, future analyses should incorporate this method for improved accuracy.

### The researchers’ measurements are consistent in our study, supporting the reliability of the findings

Next to the agreement between echocardiographic techniques, the variability between the investigators who examined the parameters is crucial when assessing echocardiography’s reproducibility in an animal model. For most systolic function and volumetric parameters, the correlation between the researchers’ observations in our study was very strong, indicating minimal interobserver variability, which was not the case using B-mode. These findings further confirm that B-mode echocardiography, which relies on geometrical assumptions, is less appropriate for evaluating DOX-induced cardiotoxicity. The study of Stegmann *et al.*^25^ showed an excellent agreement for interobserver measurements with 4DE, confirming our results, and showed a higher interobserver reproducibility with 4DE. In addition, our findings are consistent with a study investigating the reproducibility of echocardiographic techniques for assessing cardiac function in breast cancer patients undergoing chemotherapy. Indeed, Thavendiranathan *et al.*^34^ compared 2D Simpson’s method with multidimensional echocardiography (3DE) and tested the interobserver variability in assessing LVEF and volumes. In line with our results, multidimensional echocardiography showed significantly lower variability in time than the other methods. Moreover, the interobserver variability was the lowest for multidimensional echocardiography, indicating that 3DE/4DE is the most reproducible technique for LV systolic function and volume measurements. Finally, in patients with cardiovascular diseases, multidimensional echocardiography showed good interobserver variability in assessing LV function and dimensions.^35^ Therefore, 3DE/4DE of LVEF can reduce interobserver variability in patients undergoing cancer therapy and rodent models of cardiotoxicity.^36^ These findings underscore the superiority of 4D imaging as the optimal approach for obtaining reliable results in preclinical DOX-induced cardiotoxicity studies, even when different researchers perform the scans.

### Clinical relevance of the study

The functional and morphological changes following anthracycline chemotherapy resemble that of the LV dilated cardiomyopathy, characterized by dilation and a progressive decline of systolic dysfunction.^37^ This phenotype, together with an increased interstitial fibrosis, was also shown in our animal model.^9^

Regarding our animal model, the rats received repeated doses of DOX, the most commonly used anthracycline drug known for its association with cardiotoxicity, for multiple weeks to gradually develop myocardial changes over time and mimic the chronic nature of cardiotoxicity. In addition, the intravenous injection route contributes to our animal model’s translatability as it mirrors clinical protocols and results in pharmacokinetics like in patients.^38^ In contrast, most preclinical studies include a rodent model in which DOX is administered via peritoneal injections as did by O’Connell *et al.* and Hayward *et al.*, which is inconsistent with the treatment in cancer patients. This results in a lack of reproducibility and substantial variation among different studies.^39^ Finally, the cumulative DOX dose of 16 mg/kg in our study equals the clinical dose of 592 mg/m², which is administered to patients with advanced cancer and is in line with the doses used in other preclinical studies.^40^

### Limitations of the study

This study includes some limitations. First, the healthy rats used do not fully replicate the complex pathophysiological conditions seen in cancer patients with DOX-induced cardiotoxicity and multiple comorbidities. Future research should involve a tumour-induced rat model to validate our findings. Second, echocardiographic measurements were performed 8 weeks after the initial DOX injection. Long-term studies should tightly monitor changes in LV function and volumes as cardiotoxicity may still worsen even after treatment cessation. Moreover, the reproducibility of the different echocardiographic modes is only measured immediately after the last DOX injection. A temporal variability test would better capture the accuracy over time.^34^

## Conclusion

We conclude that LV volumetric changes were more pronounced than systolic dysfunction in our rat model of DOX-induced cardiotoxicity. In addition, 4DE has shown to be an effective approach, while B-mode appears less suitable. These findings underscore the importance of applying appropriate methods for detecting DOX-induced cardiotoxicity in rodent models which is crucial for testing potential cardioprotective agents before translation to larger models or human studies.

## Supplementary Material

qyaf006_Supplementary_Data

## Data Availability

No new data were generated or analysed in support of this research.

## Lead author biography

Prof. Virginie Bito obtained her PhD in Biomedical Sciences from KU Leuven (Belgium) in 2004. Her doctoral research, titled "Mechanisms of excitation-contraction coupling in myocytes from normal and chronically ischemic pig myocardium”, explored the fundamental mechanisms underlying cardiac remodelling in chronic ischaemia. Following 8 years of post-doctoral research, she was appointed associate professor at Hasselt University in 2012. With no pre-existing infrastructure or expertise in fundamental cardiology at the institute, she established her own research group from the ground up, pioneering this new domain within the university. Initially embedded within the *Physiology* research group, she is now a core member of the *Cardiac and Organ Systems* research group at BIOMED. Prof. Bito's research focuses on developing and characterizing novel therapeutic strategies to enhance cardiac function in pathological conditions such as myocardial infarction, diabetes, and anthracycline-induced cardiotoxicity. Her work includes interventions such as exercise therapy, anti-AGE therapies, and stem cell-based approaches. With a strong emphasis on fundamental research, she investigates molecular and cellular mechanisms of heart failure using preclinical animal models. She currently leads a research team of five members, supervising four PhD students-two in Belgium and two in the Democratic Republic of Congo (DRC)-alongside one post-doctoral researcher. In addition to her scientific research, she is actively involved in teaching at Hasselt University, contributing to courses for medical, biomedical, and biology students at both bachelor's and master's levels. Prof. Bito plays an important role in international academic collaboration, particularly through VLIR-UOS projects. She leads a VLIR-IUC sub-project with the Université Moulay Ismail (Morocco), serves as the promoter of a VLIR-TEAM project with the Université de Kindu (DRC), and coordinates a large VLIR-IUC project with the University of Lubumbashi (DRC). Beyond her research and academic leadership, Prof. Bito has held key administrative roles at UHasselt. Since 2013, she has chaired the Ethical Committee for Animal Experimentation, ensuring responsible and ethical research practices. In 2020, she was appointed Vice-Director of BIOMED, further contributing to the strategic development of the institute. Through her leadership in research, education, and international collaboration, Prof. Bito continues to make significant contributions to the fields of cardiovascular science and global health partnerships.

## References

[1] Wallace KB, Sardão VA, Oliveira PJ. Mitochondrial determinants of doxorubicin-induced cardiomyopathy. Circ Res 2020;126:926–41. 32213135 10.1161/CIRCRESAHA.119.314681 PMC7121924

[2] Swain SM, Whaley FS, Ewer MS. Congestive heart failure in patients treated with doxorubicin: a retrospective analysis of three trials. Cancer 2003;97:2869–79. 12767102 10.1002/cncr.11407

[3] Ewer MS, Ewer SM. Cardiotoxicity of anticancer treatments. Nat Rev Cardiol 2015;12:547–58. 25962976 10.1038/nrcardio.2015.65

[4] Omland T, Heck SL, Gulati G. The role of cardioprotection in cancer therapy cardiotoxicity: JACC: CardioOncology state-of-the-art review. JACC CardioOncol 2022;4:19–37. 35492815 10.1016/j.jaccao.2022.01.101 PMC9040117

[5] Bikiewicz A, Banach M, von Haehling S, Maciejewski M, Bielecka-Dabrowa A. Adjuvant breast cancer treatments cardiotoxicity and modern methods of detection and prevention of cardiac complications. ESC Heart Fail 2021;8:2397–418. 33955207 10.1002/ehf2.13365 PMC8318493

[6] Plana JC, Galderisi M, Barac A, Ewer MS, Ky B, Scherrer-Crosbie M et al Expert consensus for multimodality imaging evaluation of adult patients during and after cancer therapy: a report from the American Society of Echocardiography and the European Association of Cardiovascular Imaging. Eur Heart J Cardiovasc Imaging 2014;15:1063–93. 25239940 10.1093/ehjci/jeu192 PMC4402366

[7] Čelutkienė J, Plymen CM, Flachskampf FA, de Boer RA, Grapsa J, Manka R et al Innovative imaging methods in heart failure: a shifting paradigm in cardiac assessment. Position statement on behalf of the Heart Failure Association of the European Society of Cardiology. Eur J Heart Fail 2018;20:1615–33. 30411833 10.1002/ejhf.1330

[8] Asnani A, Moslehi JJ, Adhikari BB, Baik AH, Beyer AM, de Boer RA et al Preclinical models of cancer therapy–associated cardiovascular toxicity: a scientific statement from the American heart association. Circ Res 2021;129:e21–34. 33934611 10.1161/RES.0000000000000473 PMC8423100

[9] Haesen S, Jager MM, Brillouet A, de Laat I, Vastmans L, Verghote E et al Pyridoxamine limits cardiac dysfunction in a rat model of doxorubicin-induced cardiotoxicity. Antioxidants (Basel) 2024;13:112. 38247537 10.3390/antiox13010112 PMC10812466

[10] Cardinale D, Colombo A, Bacchiani G, Tedeschi I, Meroni CA, Veglia F et al Early detection of anthracycline cardiotoxicity and improvement with heart failure therapy. Circulation 2015;131:1981–8. 25948538 10.1161/CIRCULATIONAHA.114.013777

[11] Houser SR, Margulies KB, Murphy AM, Spinale FG, Francis GS, Prabhu SD et al Animal models of heart failure: a scientific statement from the American Heart Association. Circ Res 2012;111:131–50. 22595296 10.1161/RES.0b013e3182582523

[12] Fawzy AA, El-Menyawi KA, Sallam WM, Zahran ME. Two-dimensional speckle tracking echocardiography in chemotherapy-induced cardiotoxicity in females with breast cancer. Cardiooncology 2024;10:13. 38429850 10.1186/s40959-024-00209-1 PMC10905860

[13] Thavendiranathan P, Poulin F, Lim K-D, Plana JC, Woo A, Marwick TH. Use of myocardial strain imaging by echocardiography for the early detection of cardiotoxicity in patients during and after cancer chemotherapy: a systematic review. J Am Coll Cardiol 2014;63:2751–68. 24703918 10.1016/j.jacc.2014.01.073

[14] Oikonomou EK, Kokkinidis DG, Kampaktsis PN, Amir EA, Marwick TH, Gupta D et al Assessment of prognostic value of left ventricular global longitudinal strain for early prediction of chemotherapy-induced cardiotoxicity: a systematic review and meta-analysis. JAMA Cardiol 2019;4:1007–18. 31433450 10.1001/jamacardio.2019.2952 PMC6705141

[15] Zacchigna S, Paldino A, Falcão-Pires I, Daskalopoulos EP, Dal Ferro M, Vodret S et al Towards standardization of echocardiography for the evaluation of left ventricular function in adult rodents: a position paper of the ESC working group on myocardial function. Cardiovasc Res 2021;117:43–59. 32365197 10.1093/cvr/cvaa110

[16] Mor-Avi V, Lang RM, Badano LP, Belohlavek M, Cardim NM, Derumeaux G et al Current and evolving echocardiographic techniques for the quantitative evaluation of cardiac mechanics: ASE/EAE consensus statement on methodology and indications endorsed by the Japanese Society of Echocardiography. Eur J Echocardiogr 2011;12:167–205. 21385887 10.1093/ejechocard/jer021

[17] Narayan HK, French B, Khan AM, Plappert T, Hyman D, Bajulaiye A et al Noninvasive measures of ventricular-arterial coupling and circumferential strain predict cancer therapeutics-related cardiac dysfunction. JACC Cardiovasc Imaging 2016;9:1131–41. 27085442 10.1016/j.jcmg.2015.11.024 PMC5055405

[18] Chan BY, Roczkowsky A, Cho WJ, Poirier M, Sergi C, Keschrumrus V et al MMP inhibitors attenuate doxorubicin cardiotoxicity by preventing intracellular and extracellular matrix remodelling. Cardiovasc Res 2021;117:188–200. 31995179 10.1093/cvr/cvaa017 PMC7797218

[19] Kang Y, Wang W, Zhao H, Qiao Z, Shen X, He B. Assessment of subclinical doxorubicin-induced cardiotoxicity in a rat model by speckle-tracking imaging. Arq Bras Cardiol 2017;109:132–9. 10.5935/abc.20170097 PMC5576117 28700019

[20] Xiang P, Deng HY, Li K, Huang G-Y, Chen Y, Tu L et al Dexrazoxane protects against doxorubicin-induced cardiomyopathy: upregulation of Akt and Erk phosphorylation in a rat model. Cancer Chemother Pharmacol 2009;63:343–9. 18379782 10.1007/s00280-008-0744-4

[21] Bertinchant J, Polge A, Juan J, Oliva-Lauraire M, Giuliani I, Marty-Double C et al Evaluation of cardiac troponin I and T levels as markers of myocardial damage in doxorubicin-induced cardiomyopathy rats, and their relationship with echocardiographic and histological findings. Clin Chim Acta 2003;329:39–51. 12589964 10.1016/s0009-8981(03)00013-5

[22] Ohlig J, Henninger C, Zander S, Merx M, Kelm M, Fritz G. Rac1-mediated cardiac damage causes diastolic dysfunction in a mouse model of subacute doxorubicin-induced cardiotoxicity. Arch Toxicol 2018;92:441–53. 28710503 10.1007/s00204-017-2017-7

[23] Chakouri N, Farah C, Matecki S, Amedro P, Vincenti M, Saumet L et al Screening for in-vivo regional contractile defaults to predict the delayed doxorubicin cardiotoxicity in juvenile rat. Theranostics 2020;10:8130–42. 32724462 10.7150/thno.47407 PMC7381739

[24] Boyd A, Stoodley P, Richards D, Hui R, Harnett P, Vo K et al Anthracyclines induce early changes in left ventricular systolic and diastolic function: a single centre study. PLoS One 2017;12:e0175544. 28407011 10.1371/journal.pone.0175544 PMC5391073

[25] Stegmann H, Bäuerle T, Kienle K, Dittrich S, Alkassar M. 4D cardiac magnetic resonance imaging, 4D and 2D transthoracic echocardiography: a comparison of in-vivo assessment of ventricular function in rats. Lab Anim 2019;53:169–79. 30081741 10.1177/0023677218789971

[26] O’Riordan CE, Trochet P, Steiner M, Fuchs D. Standardisation and future of preclinical echocardiography. Mamm Genome 2023;34:123–55. 37160810 10.1007/s00335-023-09981-4

[27] Folland E, Parisi A, Moynihan P, Jones DR, Feldman CL, Tow D. Assessment of left ventricular ejection fraction and volumes by real-time, two-dimensional echocardiography. A comparison of cineangiographic and radionuclide techniques. Circulation 1979;60:760–6. 476879 10.1161/01.cir.60.4.760

[28] Grune J, Blumrich A, Brix S, Jeuthe S, Drescher C, Grune T et al Evaluation of a commercial multi-dimensional echocardiography technique for ventricular volumetry in small animals. Cardiovasc Ultrasound 2018;16:1–13. 29966517 10.1186/s12947-018-0128-9 PMC6029342

[29] Walker J, Bhullar N, Fallah-Rad N, Lytwyn M, Golian M, Fang T et al Role of three-dimensional echocardiography in breast cancer: comparison with two-dimensional echocardiography, multiple-gated acquisition scans, and cardiac magnetic resonance imaging. J Clin Oncol 2010;28:3429–36. 20530277 10.1200/JCO.2009.26.7294

[30] Pickett CA, Cheezum MK, Kassop D, Villines TC, Hulten EA. Accuracy of cardiac CT, radionucleotide and invasive ventriculography, two-and three-dimensional echocardiography, and SPECT for left and right ventricular ejection fraction compared with cardiac MRI: a meta-analysis. Eur Heart J Cardiovasc Imaging 2015;16:848–52. 25736307 10.1093/ehjci/jeu313

[31] Stankovic I, Dweck MR, Marsan NA, Bergler-Klein J, Holte E, Manka R et al The EACVI survey on cardiac imaging in cardio-oncology. Eur Heart J Cardiovasc Imaging 2021;22:367–71. 32464650 10.1093/ehjci/jeaa111

[32] Bunting KV, Steeds RP, Slater K, Rogers JK, Gkoutos GV, Kotecha D. A practical guide to assess the reproducibility of echocardiographic measurements. J Am Soc Echocardiogr 2019;32:1505–15. 31653530 10.1016/j.echo.2019.08.015

[33] Chen R, Zhu M, Sahn DJ, Ashraf M. Non-invasive evaluation of heart function with four-dimensional echocardiography. PLoS One 2016;11:e0154996. 27144844 10.1371/journal.pone.0154996 PMC4856388

[34] Thavendiranathan P, Grant AD, Negishi T, Plana JC, Popović ZB, Marwick TH. Reproducibility of echocardiographic techniques for sequential assessment of left ventricular ejection fraction and volumes: application to patients undergoing cancer chemotherapy. J Am Coll Cardiol 2013;61:77–84. 23199515 10.1016/j.jacc.2012.09.035

[35] Jenkins C, Bricknell K, Chan J, Hanekom L, Marwick TH. Comparison of two-and three-dimensional echocardiography with sequential magnetic resonance imaging for evaluating left ventricular volume and ejection fraction over time in patients with healed myocardial infarction. Am J Cardiol 2007;99:300–6. 17261386 10.1016/j.amjcard.2006.08.026

[36] Frey MK, Bergler-Klein J. Echocardiographic evaluation of patients undergoing cancer therapy. Eur Heart J Cardiovasc Imaging 2021;22:375–82. 33393591 10.1093/ehjci/jeaa341

[37] Jefferies JL, Towbin JA. Dilated cardiomyopathy. Lancet 2010;375:752–62. 20189027 10.1016/S0140-6736(09)62023-7

[38] Johansen PB . Doxorubicin pharmacokinetics after intravenous and intraperitoneal administration in the nude mouse. Cancer Chemother Pharmacol 1981;5:267–70. 7261254 10.1007/BF00434396

[39] Podyacheva EY, Kushnareva EA, Karpov AA, Toropova YG. Analysis of models of doxorubicin-induced cardiomyopathy in rats and mice. A modern view from the perspective of the pathophysiologist and the clinician. Front Pharmacol 2021;12:670479. 34149423 10.3389/fphar.2021.670479 PMC8209419

[40] Nair AB, Jacob S. A simple practice guide for dose conversion between animals and human. J Basic Clin Pharm 2016;7:27–31. 27057123 10.4103/0976-0105.177703 PMC4804402

