# Supplementary material for: Comprehensive transthoracic echocardiographic evaluation of doxorubicin-induced cardiotoxicity: a multimodal imaging approach in an animal model
Source: Eur Heart J Imaging Methods Pract. 2025 Jan 10;3(1):qyaf006. doi: 10.1093/ehjimp/qyaf006 (PMC11837189; doi:10.1093/ehjimp/qyaf006)
Supplement: qyaf006_Supplementary_Data [file qyaf006_supplementary_data.docx]

**Supplementary Tables**

**Table S1: Echocardiographic parameters of LV systolic function at baseline, week 4, 6, and 8.**

|  | **CTRL** | **DOX** |
| --- | --- | --- |
| **LVEF (%)** |  |  |
| Baseline | 87.05 ± 0.93 | 87.30 ± 1.27 |
| Week 4 | 83.92 ± 1.13 | 81.01 ± 3.07 |
| Week 6 | 88.00 ± 1.18 | 81.62 ± 2.63 ^*^ |
| Week 8 | 86.03 ± 1.08 | 61.00 ± 3.30 ^****, ####, ‡‡‡‡^ |
| **Longitudinal LVFS (%)** |  |  |
| Baseline | 23.63 ± 1.16 | 26.15 ± 0.83 |
| Week 4 | 23.03 ± 1.69 | 22.34 ± 2.95 |
| Week 6 | 29.99 ± 2.46 | 25.18 ± 1.77 |
| Week 8 | 26.72 ± 0.84 | 14.74 ± 0.89 ^****, ####, ‡‡‡‡^ |
| **Radial LVFS (%)** |  |  |
| Baseline | 54.51 ± 1.76 | 54.10 ± 1.84 |
| Week 4 | 60.83 ± 2.74 | 57.56 ± 1.48 |
| Week 6 | 53.48 ± 1.88 ^‡^ | 46.38 ± 1.76 ^*, †^ |
| Week 8 | 55.81 ± 1.45 ^‡^ | 33.26 ± 2.10 ^****, #### , ‡‡‡‡^ |
| **LV cardiac index (mL/min/cm^2^)** |  |  |
| Baseline | 1.16 ± 0.01 | 0.18 ± 0.01 |
| Week 4 | 0.17 ± 0.01 | 0.18 ± 0.01 |
| Week 6 | 0.18 ± 0.01 ^‡^ | 0.21 ± 0.01 |
| Week 8 | 0.14 ± 0.01 | 0.13 ± 0.01 ^***, ‡‡‡‡^ |

LV echocardiographic parameters for systolic function were measured in CTRL (N = 14) and DOX (N = 14) animals at baseline and after four, six, and eight weeks of DOX injections. Data are presented as mean ± SEM. ^*^P<0.05, ^***^P<0.001, and ^****^P<0.0001 vs. baseline. ^####^P<0.0001 vs. CTRL. ^†^P<0.05 vs. week 4. ^‡^P<0.05 and ^‡‡‡‡^P<0.0001 vs. week 6. CTRL, control. DOX, doxorubicin. LV, left ventricular. LVEF, LV ejection fraction. LVFS, LV fractional shortening.

**Table S2. Echocardiographic parameters of LV volumes at baseline, week 4, 6, and 8.**

|  | **CTRL** | **DOX** |
| --- | --- | --- |
| **LVESV/BSA (µl/cm^2^)** |  |  |
| Baseline | 0.07 ± 0.007 | 0.08 ± 0.009 |
| 4W | 0.08 ± 0.004 | 0.11 ± 0.017 |
| 6W | 0.07 ± 0.006 | 0.14 ± 0.219 ^**, #^ |
| 8W | 0.07 ± 0.007 | 0.30 ± 0.035 ^****, ####^ |
| **LVEDV/BSA (µl/cm^2^)** |  |  |
| Baseline | 0.55 ± 0.02 | 0.59 ± 0.02 |
| 4W | 0.48 ± 0.01 | 0.58 ± 0.02 |
| 6W | 0.65 ± 0.01 ^†^ | 0.77 ± 0.03 ^****, ##^ |
| 8W | 0.50 ± 0.01 ^‡‡^ | 0.75 ± 0.04 ^****, ##^ |

LV volumes were measured in CTRL (N = 14) and DOX (N = 14) animals at baseline and after four, six, and eight weeks of DOX injections. Data are presented as mean ± SEM. ^**^P<0.01 and ^****^P<0.0001 vs. baseline. ^#^P<0.05, ^##^P<0.01, and ^####^P<0.0001 vs. CTRL. ^†^P<0.05 vs. week 4. ^‡‡^P<0.01 vs. week 6. BSA, body surface area. CTRL, control. DOX, doxorubicin. LV, left ventricular. LVEDV, LV end-diastolic volume. LVEF, LV ejection fraction. LVESV, LV end-systolic volume.

**Supplementary Figures**

**
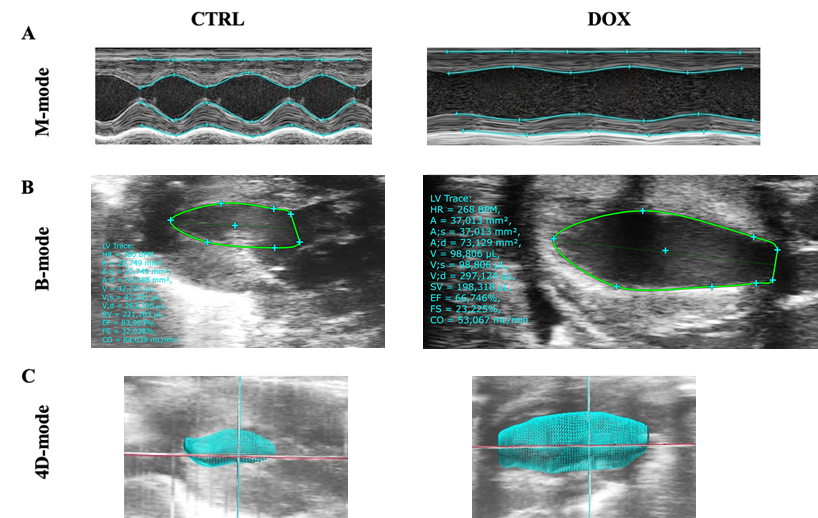
**

**Figure S1. Representative echocardiographic images obtained at week 8. (A-C)** Representative images of M-mode **(A)**, B-mode during systole **(B)**, and 4D reconstructed hearts during systole **(C)** at week 8 in CTRL and DOX animals.


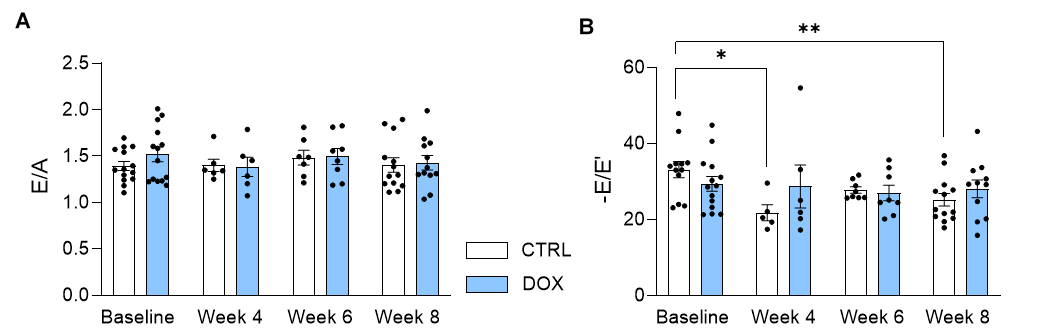


**Figure S2. LV diastolic function over time.** E/A **(A)** and −E/E’ **(B)** measured in CTRL (N=14) and DOX (N=14) animals. Data are shown as mean ± SEM. For CTRL: N=14 (baseline), N=13 (week 8), N=6 (week 4), and N=7 (week 6). For DOX: N=14 (baseline), N=12 (week 8), N=6 (week 4), and N=8 (week 6). ^*^P<0.05, ^**^P<0.01. E/A, the ratio of peak flow velocity in early versus late filling. CTRL, control. DOX, doxorubicin. E/E’, the ratio of peak mitral flow versus annular velocity.

**Figure S3. Survival.** Survival for CTRL and DOX groups represented as a Kaplan-Meier plot. CTRL, control DOX, doxorubicin.


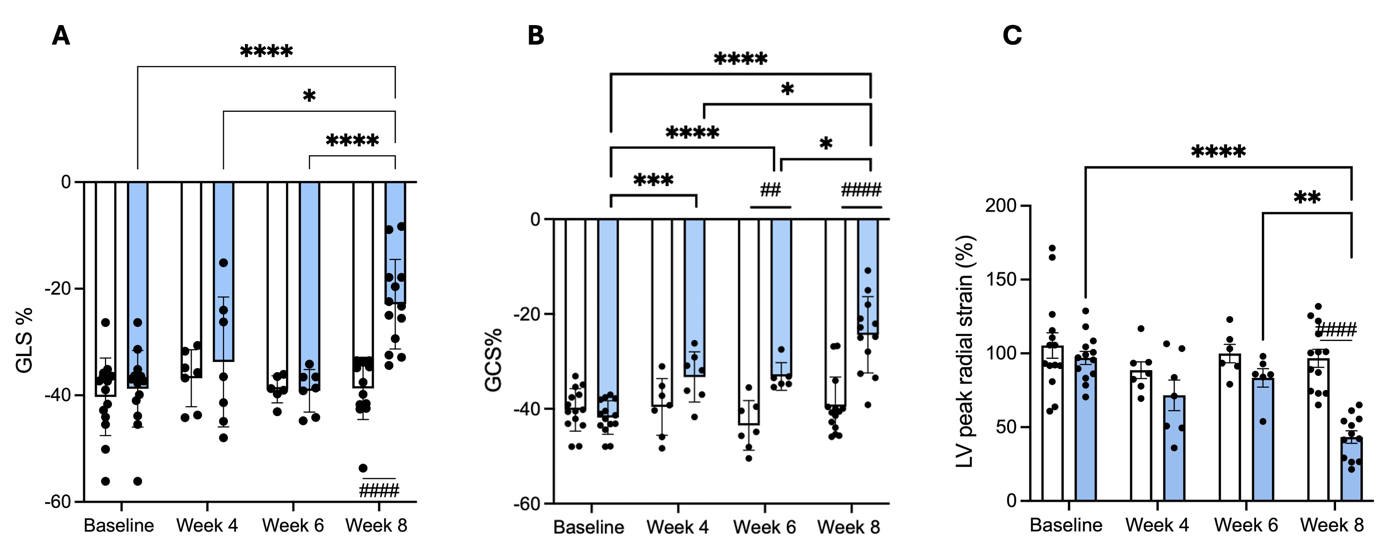


**Figure S4. LV strain measurement over time.** GLS **(A)**, GCS **(B)**, and LV peak radial strain **(C)** were measured in CTRL and DOX animals (both N=14). Data are shown as mean ± SEM. For CTRL: N=14 (baseline, week 8) and N=7 (week 4, week 6). For DOX: N=14 (baseline, week 8), N=4/6 (week 4), and N=8 (week 6). ^*^P<0.05, ^**^P<0.01, ^***^P<0.001, ^****^P<0.0001. ^##^P<0.01, ^####^P<0.0001. CTRL, control. DOX, doxorubicin. GCS, global circumferential strain. GLS, global longitudinal strain. LV, left ventricular.
